# Tau pathology in the retina and its role in neurodegeneration

**DOI:** 10.1002/alz70855_102036

**Published:** 2025-12-23

**Authors:** Dietmar Rudolf Thal, Alicja Ronisz, Simona Ospitalieri, Rita Van Ginderdeuren, Grigoria Tsaka, Sandra O. Tomé, Sophie Lemmens, Ingeborg Stalmans, Rik Vandenberghe, Christine A.F. von Arnim, Joost Schymkowitz, Frederic Rousseau, Jeroen J.M. Hoozemans, Annemieke J.M. Rozemuller, Frederique J. Hart de Ruyter, Tjado Morrema, Femke H. Bouwman, Lies De Groef, Grzegorz Walkiewicz

**Affiliations:** ^1^ Laboratory of Neuropathology, KU Leuven, Leuven, Vlaams‐Brabant, Belgium; ^2^ University Hospital Leuven, Department of Pathology, Leuven, Belgium; ^3^ Leuven Brain Institute, Leuven, Belgium; ^4^ Laboratory of Neuropathology, KU Leuven, Leuven, Vlaams Brabant, Belgium; ^5^ University Hospital Leuven, Leuven, Vlaams‐Brabant, Belgium; ^6^ Switch laboratory, VIB ‐ KU Leuven, Leuven, Belgium; ^7^ University Hospital Leuven, Leuven, Belgium; ^8^ Research Group Ophthalmology, Leuven Brain Institute, KU Leuven, Leuven, Vlaams‐Brabant, Belgium; ^9^ University Hospitals Leuven, Leuven, Belgium; ^10^ Laboratory for Cognitive Neurology, KU Leuven, Leuven, Leuven, Belgium; ^11^ Georg‐August‐University, Goettingen, Niedersachsen, Germany; ^12^ University of Ulm, Ulm, Germany; ^13^ Switch laboratory, VIB ‐ KU Leuven Center for Brain & Disease Research, Leuven, Vlaams Brabant, Belgium; ^14^ Switch Laboratory, VIB ‐ KU Leuven Center for Brain & Disease Research, Leuven, Vlaams‐Brabant, Belgium; ^15^ Amsterdam UMC, location VUmc, Amsterdam, na, Netherlands; ^16^ Department of Pathology, Amsterdam Neuroscience, Amsterdam UMC, Amsterdam, Noord‐Holland, Netherlands; ^17^ Department of Pathology, Amsterdam Neuroscience, Amsterdam UMC, Amsterdam, na, Netherlands; ^18^ Department of Neurology and Alzheimer Center Amsterdam, Amsterdam Neuroscience, VU University Medical Center, Amsterdam UMC, Amsterdam, The Netherlands, Amsterdam, Netherlands; ^19^ Cellular Communication and Neurodegeneration Research Group, Leuven Brain Institute, KU Leuven, Leuven, Vlaams‐Brabant, Belgium

## Abstract

The accumulation of abnormal phosphorylated tau (*p*‐τ) protein in neurons is a hallmark of Alzheimer's disease (AD) and primary tauopathies. Both conditions involve *p*‐τ accumulation in retinal neurons. Here, we explore the role of *p*‐τ pathology in visual performance and the interplay between retinal and cerebral *p*‐τ pathology.

In the human retina, *p*‐τ is frequently found. This retinal tauopathy evolves in three stages: stage 1 involves *p*‐τ aggregates in the outer plexiform layer, stage 2 affects neurons in the inner nuclear layer, and stage 3 involves the inner plexiform layer. Various phosphorylation sites (S202/T205, T231, S396/404) and conformational τ epitopes are present in human retinal tauopathy, but no argyrophilic fibrils are seen. Western blot analysis shows a distinct molecular pattern of *p*‐τ species in the retina compared to cerebral tauopathies like AD and primary age‐related tauopathy (PART). The stages of this primary retinal tauopathy (PReT) correlate with altered visual performance and dementia symptoms.

In transgenic mouse models, *p*‐τ is prominent in retinal ganglion cells, which can develop fibrillar aggregates, unlike in human retinal tauopathy. In the TAU58 mouse model, tauopathic changes in the retina are associated with ganglion cell loss. TAU58 mice show enhanced tau pathology in the retina after AD brain lysate injection, with increased tau changes in the contralateral superior colliculus, suggesting propagation from retina to brain. Lysates from a Braak stage I PART case did not induce propagation but accelerated seeding in the retina.

These findings suggest that human PReT, with its low levels of *p*‐τ and lack of fibrillar *p*‐τ lesions, is a separate entity from AD, especially in young individuals. However, propagation of *p*‐τ pathology from retina to brain is possible. PReT's association with visual worsening in humans and reduced retinal ganglion cells in mice needs further exploration. PReT may also be a prerequisite for retinal involvement in AD and primary tauopathies, as indicated by its association with dementia symptoms.

**Funding**: SAO/FRA 2020/017

